# Upregulated UHRF1 Promotes Bladder Cancer Cell Invasion by Epigenetic Silencing of KiSS1

**DOI:** 10.1371/journal.pone.0104252

**Published:** 2014-10-01

**Authors:** Yu Zhang, Zhen Huang, Zhiqiang Zhu, Xin Zheng, Jianwei Liu, Zhiyou Han, Xuetao Ma, Yuhai Zhang

**Affiliations:** 1 Department of Urology, Beijing Friendship Hospital, Capital Medical University, Beijing, China; 2 Department of Urology, Beijing You An Hospital, Capital Medical University, Beijing, China; 3 Department of Urology, Dongzhimen Hospital Affiliated to Beijing University of Chinese Medicine, Beijing, China; Louisiana State University Health Sciences center, United States of America

## Abstract

Ubiquitin-like with PHD and RING finger domains 1 (UHRF1), as an epigenetic regulator, plays important roles in the tumorigenesis and cancer progression. KiSS1 functions as a metastasis suppressor in various cancers, and epigenetic silencing of KiSS1 increases the metastatic potential of cancer cells. We therefore investigated whether UHRF1 promotes bladder cancer cell invasion by inhibiting KiSS1. The expression levels of UHRF1 and KiSS1 were examined by quantitative real-time PCR assay *in vitro* and *in vivo*. The role of UHRF1 in regulating bladder cancer metastasis was evaluated in bladder cancer cell. We found that UHRF1 levels are upregulated in most clinical specimens of bladder cancer when compared with paired normal tissues, and UHRF1 expression levels are significantly increased in primary tumors that subsequently metastasized compared with non-metastatic tumors. Forced expression of UHRF1 promotes bladder cancer cell invasion, whereas UHRF1 knockdown decreases cell invasion. Overexpression of UHRF1 increases the methylation of CpG nucleotides and reduces the expression of KiSS1. UHRF1 and KiSS1 expression level is negatively correlated *in vivo* and *in vitro*. Knockdown of KiSS1 promotes bladder cancer cell invasion. Importantly, forced expression of KiSS1 partly abrogates UHRF1-induced cell invasion. These data demonstrated that upregulated UHRF1 increases bladder cancer cell invasion by epigenetic silencing of KiSS1.

## Introduction

Human bladder cancer ranks second in frequency of genitourinary cancer [1], and approximately 50% of patients diagnosed with muscle-invasive bladder cancer (MIBC) develop distant metastases in the lungs and liver, resulting in poor 5-year survival rates [2]. Currently, the advances in suitable therapy for enhancing survival rate are limited because the underlying mechanisms causing cancer metastasis are not well understood. Therefore, it is very important to reveal the molecular mechanism of bladder cancer metastasis for developing effective therapy.

UHRF1, also called ICBP90 in humans and Np95 in mice, is a multidomain protein, which is required for epigenetic regulation of gene expression and chromatin modification [3], [4]. UHRF1 increases the G1/S transition as the target of E2F transcription factor [5], and changes in UHRF1 expression level regulates cell cycle progression, cell proliferation and cell migration [6], [7]. UHRF1 is upregulated in multiple types of cancers, and overexpression of UHRF1 is involved in tumorigenesis and cancer progression [8], [9]. Several reports showed that UHRF1 may be an important biomarker for diagnosis and prognosis of cancers [5]. Unoki *et al* demonstrated that UHRF1 overexpression is associated with the grade and stage of bladder cancer [10]. Overexpression of UHRF1 in bladder cancer is also correlated with increased risk of cancer progression after transurethral resection [10]. Wang *et al* showed that UHRF1 is overexpressed in colorectal cancer (CRC) cell lines and clinical specimens [5]. UHRF1 expression levels are correlated with cancer metastasis and poor Dukes staging. UHRF1 knockdown induces cell apoptosis and cell cycle arrest at the G0/G1 phase and suppresses cell proliferation and migration.

UHRF1 is a very important regulator of DNA methylation, and aberrant DNA methylation is a frequent epigenetic event in bladder cancer [6], [11]. UHRF1 recognizes hemimethylated DNA generated during DNA replication and recruits DNMT1 (DNA methyltransferase1) to ensure faithful maintenance of DNA-methylation patterns in daughter cells [6]. KiSS1 is identified as suppressing metastases in various cancers, such as bladder cancer, breast cancer cells and melanoma [12]. KiSS1 encodes a 145-amino acid protein that is processed into KiSSpeptins (KP), including KP10, KP13, KP14 and KP54 [12], [13], [14]. Recent studies showed that KiSS1 is epigenetically silenced by hypermethylation in bladder cancer [15]. Cebrian *et al* demonstrated that KiSS1 hypermethylation is frequently observed and is correlated with low gene expression, being restored by demethylating azacytidine in bladder cancer cells [12]. Hypermethylation of KiSS1 is also correlated with high tumor grade and stage. Despite these intriguing findings, little is known about whether UHRF1 increases bladder cancer cell invasion by epigenetic silencing of KiSS1.

In the study, we tested whether UHRF1/KiSS1 represents a novel pathway regulating bladder cancer cell invasion. Our results revealed that UHRF1 expression is upregulated in primary tumors that subsequently metastasized, and overexpression of UHRF1 promotes bladder cancer cell invasion by epigenetic silencing of KiSS1.

## Materials and Methods

### Clinical samples and cell lines

Human bladder tissues were obtained with written informed consent from the Beijing Friendship Hospital affiliated to Capital Medical University. The study was approved by the Ethics Committee of Capital Medical University. 47 specimens (Table 1) of pathologically and normally diagnosed biopsy specimens (≥3 cm away from bladder cancer tissues) were collected from patients with bladder cancer, including 22 with non-muscle-invasive [NMI, stage pTa-pT1] and 25 with muscle-invasive [MI, stage ≥T2].Human bladder cancer cells were obtained from the American Type Culture Collection (ATCC, Manassas, VA) and were maintained in RPMI 1640 with 10% FBS (GIBCO, Carlsbad, CA).

**Table 1:** Associations of UHRF1 level with clinicopathologic characteristics in 47 patients with bladder cancer.

Variable	n	UHRF1 level		*P*
		low	high	
**Age**	47			
*<60*	19	8	11	0.594
*≥60*	28	12	16	
**Gender**				
*Male*	31	14	17	0.365
*Female*	16	6	10	
**Histologic grade**				
*G1*	8	3	5	0.058
*G2*	29	13	16	
*G3*	10	4	6	
**Depth of invasion**				
*pTa-pT1*	22	10	12	0.042
*pT2-pT4*	25	10	15	
**KiSS1 level**				
*Low*	31	14	17	0.033
*High*	16	10	6	

Fisher's exact test was used for the statistical analyses.

### Real-time quantitative PCR

Total RNA from bladder cancer cell lines and specimens was extracted using Trizol reagent (Invitrogen, Carlsbad, CA). The RT (reverse-transcription) reaction was performed using an M-MLV Reverse Transcriptase kit (Invitrogen). Real-time quantitative PCR was carried out using a standard SYBR Green PCR Master Mix (Life Technologies) protocol on the StepOne Real-Time PCR System (Applied Biosystems) according to the instructions from the respective manufacturer. β-actin was used as internal control for mRNA. Respective ΔCt values (both UHRF1 and KiSS1) were obtained by normalization to β-actin. Relative expression was calculated with respect to the control. The results were expressed as 2^−ΔΔCt^. *p<0.05.

### Overexpression and Small interfering RNA

To overexpress UHRF1, plasmid pcDNA-UHRF1 was constructed by introducing a *HindIII-EcoRI* fragment containing the UHRF1 cDNA into the same sites in pcDNA3.1. The UHRF1 gene was amplified by PCR using the forward and reverse primers: cccaagcttgggATGTGGATCCAGGTTCGGACCATGGACGGG and ggaattccTCACCGGCCATTGCCGTAGCCGGGGAAG. pcDNA-UHRF1 was transfected into bladder cancer cell lines by using Lipofectamine 2000 (Invitrogen).

To inhibit endogenous UHRF1 and KiSS1 expression, bladder cancer cells were transfected with 30 nM indicated indicated siRNA or negative control using Lipofectamine 2000. UHRF1-siRNAs (reference sequence, sc-76805) were purchased from Santa Cruz Biotechnology (Santa Cruz Biotechnology, Santa Cruz, CA). The KiSS1-siRNAs (reference sequence, sc-37443) were purchased from Santa Cruz Biotechnology.

### Transwell invasion assay

Cell invasion was examined using Transwell invasion assay with inserts of 8-µm pore size (Corning Costar) as described previously [16]. Briefly, bladder cancer cells (RT4 or T24) were suspended in serum free medium, and then seeded onto Matrigel-coated Transwell filters in Biocoat Matrigel invasion chambers. The serum-containing medium was used as a chemo-attractant in the lower chamber. The bladder cancer cells were treated with indicated reagents for 72 h, and then cells that did not invade through the pores were eliminated by using a cotton swab. Cells on the lower surface of the membrane were stained with Crystal violet. The cell numbers were determined by counting of the penetrating cells under a microscope at 200× magnification in random fields in each well.

### Methylation Analysis of *KiSS1*

A search for enrichment of CpG in KiSS1 was performed and bisulfite sequencing primers were designed using the CpG Island Searcher online tool (MethPrimer, http://www.urogene.org/methprimer/). The forward primers are GATGGAAGGGGAATAGTTTTATTAGA, and the reverse primers are TACAACTAAAACTCCTTCCACCTAC [12].

Genomic DNA was prepared from bladder cancer cells using the QiAmp DNA blood Mini kit, and then the genomic DNA was bisulfite modified using EZ DNA Methylation-Gold Kit from Zymo research [17]. The PCR products were cloned into pMD-18T (TaKaRa) according to manufacturer's instructions. 9 positive clones were sequenced. The data were analyzed using the BiQ analyzer software [17].

### Statistical Analysis

Data are expressed as mean ±SD (standard deviation) from at least three separate experiments. The differences between groups were analyzed using Student's *t* test. The difference was deemed statistically significant at *p*<0.05.

## Results

### UHRF1 level is upregulated in metastatic bladder urothelial carcinoma

**Figure 1:**
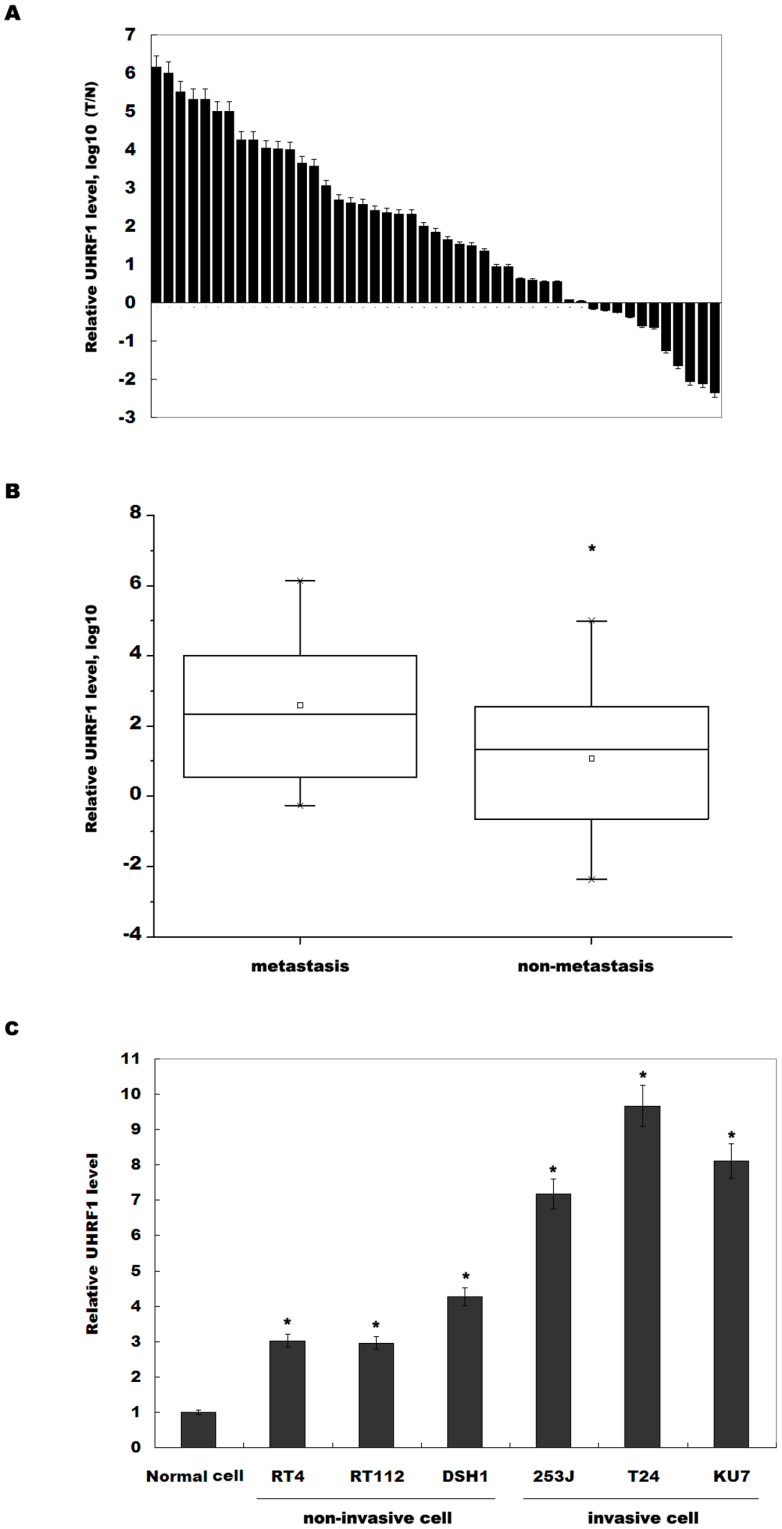
Higher UHRF1 expression is associated with bladder cancer metastasis. (A) The quantitative analysis of UHRF1 expression level was carried out in bladder cancer tissues (n = 47) and adjacent normal tissues. Total RNA was extracted and subjected to real-time PCR to analyze the expression of UHRF1 in each sample. β-actin was used as an internal control. The relative UHRF1 level was calculated by 2^−ΔΔCt^ where ΔCt = Ct (UHRF1) – Ct (β-actin) and ΔΔCt = ΔCt (tumor tissue) – ΔCt (adjacent normal tissue). (B) The bladder cancer specimens were divided into two groups based on clinical progression. The UHRF1 levels in the metastasis group (n = 25) were higher than those in the no-metastasis group (n = 22). *p<0.05. (C) UHRF1 expression level was assayed by real-time PCR in three noninvasive and three invasive bladder cancer cell lines. Normal urothelial cells were used as control. *p<0.05.

UHRF1 results in abnormal DNA methylation and cancer metastasis, and UHRF1 expression is correlated with a poor prognosis in several cancers. To assess whether UHRF1 regulates bladder cancer metastasis, we first examined UHRF1 expression in bladder cancer cell lines and cancer tissues. Figure 1A showed that the expression levels of UHRF1 are significantly upregulated in most bladder cancer tissues compared with adjacent normal controls. UHRF1 is remarkably increased expression in 77% (36/47) of bladder cancer tissues (Figure 1A). When the 47 tumor tissues were stratified based on clinical progression, UHRF1 expression is significantly increased in primary tumors that subsequently metastasized compared with non-metastatic tumors (Figure 1B). Then we assayed the expression levels of UHRF1 in both invasive bladder cancer cell lines and noninvasive bladder cancer cell lines. Among the three invasive cell lines (253J, T24 and KU7) the UHRF1 levels are relatively higher than those in the noninvasive ones (Figure 1C). These data suggest that UHRF1 overexpressoin may be related to bladder cancer metastasis.

### Upregulated UHRF1 increases bladder cancer cell invasion *in vitro*

**Figure 2:**
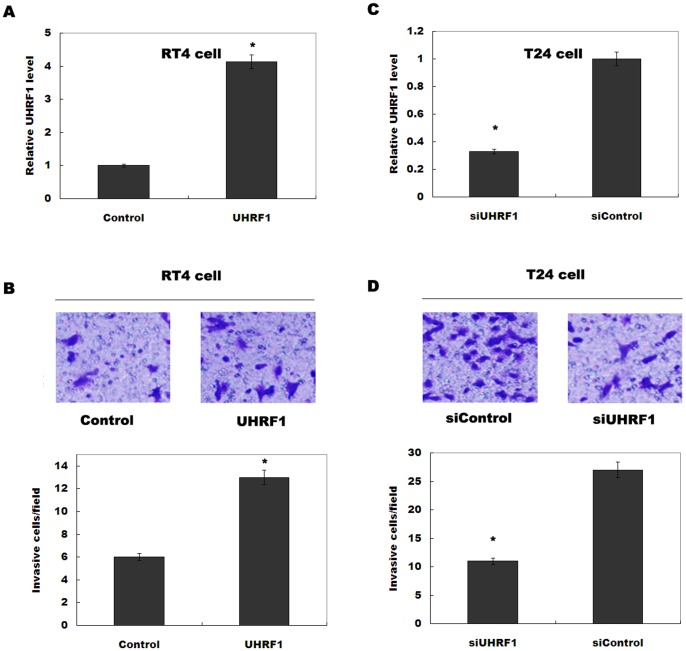
Overexpression of UHRF1 promotes bladder cancer cell invasion. (A) RT4 cells were treated with pcDNA-UHRF1, and the relative level of UHRF1 was assayed by realtime PCR. *p<0.05. (B) UHRF1 was overexpressed in RT4 cells, and invasion assay was performed as described in Material and Methods. Representative figures of each experiment are shown. These results show data from five independent experiments, expressed as the mean ±SD. *p<0.05. (C) T24 cells were treated with UHRF1-siRNA, and the relative level of UHRF1 was assayed by realtime PCR. *p<0.05. (D) Invasion assay was performed after UHRF1 knockdown. Representative figures of each experiment are shown. These results show data from five independent experiments, expressed as the mean ±SD. *p<0.05.

To investigate the role of UHRF1 in regulating cell invasion, the bladder cancer cell lines treated with UHRF1-siRNA or pcDNA-UHRF1 were analyzed. We first demonstrated whether RT4 and T24 cells can be used as *in vitro* model to investigate UHRF1 by assaying its expression in RT4 or T24 cells after overexpression or knockdown of UHRF1. Figure 2A and C showed that the pcDNA-UHRF1 treatment significantly increases UHRF1 levels in non-invasive RT4 cells, whereas UHRF1-siRNA treatment decreases UHRF1 expression in invasive T24 cells. Furthermore, upregulation of UHRF1 promotes RT4 cell invasion (Figure 2B), whereas siRNA-mediated UHRF1 silencing inhibits T24 cell invasion (Figure 2D). These results demonstrated that UHRF1 positively regulates bladder cancer cell invasion.

### UHRF1 increases the methylation of CpG nucleotides and reduces the expression of KiSS1

**Figure 3:**
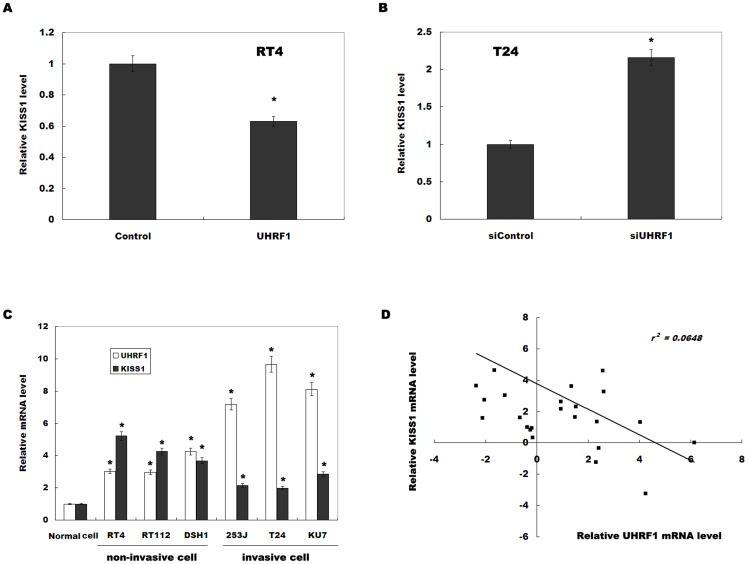
UHRF1 negatively regulates KiSS1 expression. (A and B) KiSS1 expression level was assayed in RT4 cells overexpressed with UHRF1 or T24 cells treated with UHRF1-siRNA. *p<0.05. (C) UHRF1 and KiSS1 levels were assayed by real-time PCR in three noninvasive and three invasive bladder cancer cell lines. Normal urothelial cells were used as control. *p<0.05. (D) Negative correlation between the UHRF1 mRNA levels and the KiSS1 levels in 24 bladder cancer samples (*r* ^2^ = 0.0648, *p* = 0.0063).

**Figure 4:**
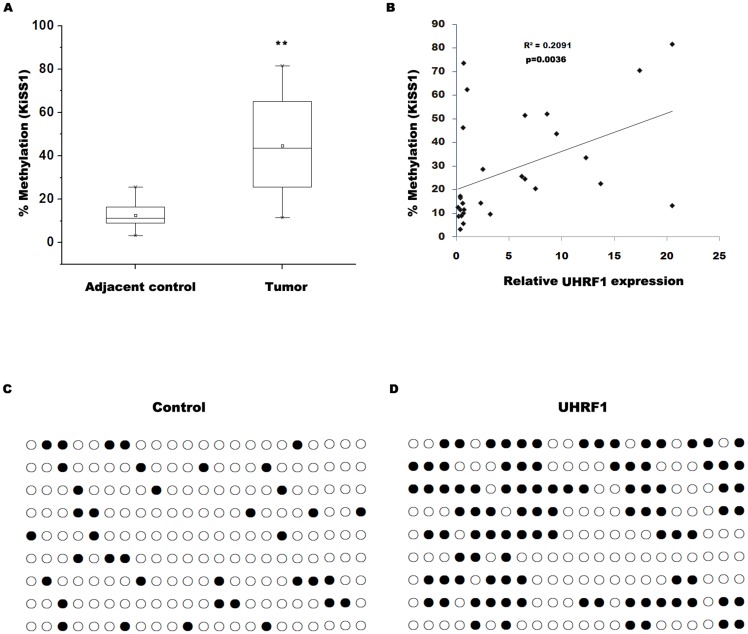
UHRF1 reduces the expression of KiSS1 by increasing the methylation of CpG nucleotides. (A) The methylation analysis of the *KiSS1* gene in 14 bladder cancer tissues and adjacent normal samples. BSQ analysis revealed that *KiSS1* methylation frequency is markedly increased with bladder cancer samples. ** p<0.01. (B) A positive correlation is observed between *KiSS1* methylation and UHRF1 expression (R^2^ = 0.2091, p = 0.0036). (C and D) CpG island methylation status of KiSS1 was analyzed by bisulfite sequencing in RT4 cells. Nine individual clones were shown per cell line. CpG dinucleotides were represented as dark squares for methylated cytosines and open squares for unmethylated cytosines.

Previous studies showed that KiSS1 is frequently hypermethylated in bladder cancer and is correlated with cancer progression [12]. Here we investigated whether UHRF1 regulates bladder cancer cell invasion by epigenetic silencing of KiSS1. We first assayed whether UHRF1 regulates KiSS1 expression. As shown in Figure 3A and B, forced expression of UHRF1 inhibits KiSS1 expression, whereas knockdown of UHRF1 increases KiSS1 expression. We then assayed the expression level of UHRF1 and KiSS1 in both invasive bladder cancer cell lines and noninvasive bladder cancer cell lines. Figure 3C showed that the UHRF1 levels are relatively higher with concurrent low levels of KiSS1 in invasive cell lines. A significant negative correlation is also observed between the UHRF1 mRNA levels and the KiSS1 mRNA levels *in vivo* (*r* ^2^ = 0.0648, *p* = 0.0063, Figure 3D). We further investigated whether UHRF1 inhibits KiSS1 expression by increasing the methylation of CpG nucleotides of *KiSS1*. We analyzed the methylation status of CpG islands in KiSS1 by bisulfite sequencing, as reported previously [12], [15]. The methylation assay results for the *KiSS1* gene in 14 bladder cancer tissues and adjacent normal controls are summarized in Figure 4A. Bisulfite sequencing analysis revealed that *KiSS1* methylation frequency is markedly increased in bladder cancer tissues compared with adjacent controls (Figure 4A). Correlation analysis showed that the *KiSS1* methylation level is positively correlated with UHRF1 expression in both bladder cancer and normal bladder tissues (Figure 4B, p = 0.0036, R^2^ = 0.2091). Bisulfite sequencing analysis further showed that UHRF1 overexpression increases the methylation of CpG nucleotides of *KiSS1* (Figure 4C,D). These results suggest that UHRF1 suppresses KiSS1 expression by epigenetic silencing of KiSS1.

### UHRF1 increases bladder cancer cell invasion by inhibiting KiSS1

**Figure 5:**
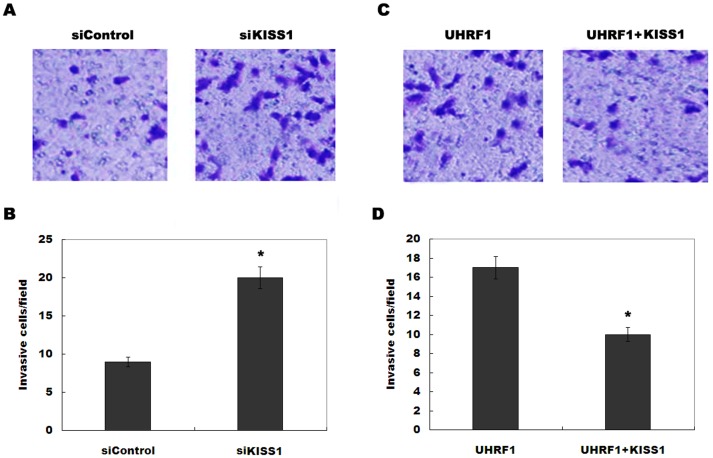
UHRF1 increases bladder cancer cell invasion by inhibiting KiSS1. (A and B) RT4 cells were treated with KiSS1-siRNA and invasion assay was performed after KiSS1 knockdown. Representative figures of each experiment are shown. These results show data from five independent experiments, expressed as the mean ±SD. *p<0.05. (C and D) RT4 cells were overexpressed with the UHRF1 or UHRF1 plus KiSS1, and invasion assay was performed. *p<0.05.

UHRF1 decreases KiSS1 expression by increasing the methylation of CpG nucleotides of *KiSS*, and downregulation of KiSS1 promotes bladder cancer cell invasion. Therefore we speculated that the role of UHRF1 in regulating cell invasion is partly mediated by KiSS1. We first assayed whether KiSS1 inhibition increases bladder cancer cell invasion. As shown in Figure 5A and B, knockdown of KiSS1 promotes RT4 cell invasion. More important, KiSS1 overexpression partly inhibits UHRF1-inducing RT4 cell invasion (Figure 5C and D). These results confirm that UHRF1 promotes bladder cancer cell invasion, at least in part, by epigenetic silencing of KiSS1.

## Discussion

In mammals, DNA methylation occurs at the C5 position of cytosine in the context of CpG dinucleotides, resulting in 5-methylcytosine (5mC) [18]. The modification of genomic DNA by methylation is an important epigenetic signal, and changes in the pattern of DNA methylation have been a consistent finding in various tumor types [19]. Recent studies showed that epigenetic inheritance of DNA methylation requires UHRF1, which recruits DNMT1 to DNA replication forks through a unique hemimethylated CpG-binding activity [20]. Liu *et al* reported that UHRF1 recruits DNMT1 for DNA maintenance methylation through binding either hemi-methylated CpG or H3K9me2/3, and that the presence of both binding activities ensures high fidelity DNA maintenance methylation [20].

UHRF1 overexpression is associated with the tumor stages and predicts poor prognoses in various cancers [21]. UHRF1 coordinates PPARG (peroxisome proliferator-activated receptor γ) epigenetic silencing and mediates colorectal cancer progression [22]. Knockdown of UHRF1 elicits PPARG re-activation, accompanied by positive histone marks and DNA demethylation, corroborating its role in PPARG silencing. Babbio *et al* showed that UHRF1 contributes to epigenetic gene silencing in prostate cancer progression [23]. In the present study, we found that the expression levels of UHRF1 are significantly increased in most bladder cancer tissues compared with adjacent normal controls. Moreover, UHRF1 expression is significantly increased in primary tumors that subsequently metastasized compared with non-metastatic tumors. We also found that the UHRF1 levels are relatively higher in invasive bladder cancer cell lines than those in the noninvasive ones. Furthermore, upregulated UHRF1 promotes noninvasive RT4 cell invasion, whereas knockdown of UHRF1 inhibits invasive T24 cell invasion. These data demonstrated that upregulation of UHRF1 contributes to bladder cancer cell invasion.

KiSS1 is a tumor metastasis suppressor gene in several cancers. KiSS1 expression is markedly decreased in invasive bladder tumors compared with their respective normal urothelium [24]. Lower KiSS1 level is correlated with overall survival in bladder tumors [24]. KiSS1 is significantly methylated. Inactivation of KiSS1 by promoter methylation is an infrequent event in pancreatic ductal adenocarcinoma (PDAC) [25]. KiSS1 is also epigenetically modified in colorectal cancer, and KiSS1 methylation is associated with tumor grade, predicted recurrence and overall survival [15]. Here we found that UHRF1 negatively regulates KiSS1 expression. UHRF1 levels are relatively higher with concurrent low levels of KiSS1 in invasive bladder cancer cell lines. A significant negative correlation is also observed between the UHRF1 mRNA levels and the KiSS1 mRNA levels *in vivo*. Bisulfite sequencing analysis showed that UHRF1 inhibits KiSS1 expression by increases the methylation of CpG nucleotides of *KiSS1*. More important, KiSS1 overexpression partly inhibits RT4 cell invasion in UHRF1-overexpressing cells.

### Conclusion

Our data showed that upregulated UHRF1 contributes to bladder cancer cell invasion by epigenetic silencing of KiSS1.
